# Jammer Localization Using Wireless Devices with Mitigation by Self-Configuration

**DOI:** 10.1371/journal.pone.0160311

**Published:** 2016-09-01

**Authors:** Qazi Mamoon Ashraf, Mohamed Hadi Habaebi, Md. Rafiqul Islam

**Affiliations:** Department of Electrical and Computer Engineering, University Islam Antarabangsa, Jalan Gombak, Selangor, Malaysia; UMIT, AUSTRIA

## Abstract

Communication abilities of a wireless network decrease significantly in the presence of a jammer. This paper presents a reactive technique, to detect and locate the position of a jammer using a distributed collection of wireless sensor devices. We employ the theory of autonomic computing as a framework to design the same. Upon detection of a jammer, the affected nodes self-configure their power consumption which stops unnecessary waste of battery resources. The scheme then proceeds to determine the approximate location of the jammer by analysing the location of active nodes as well as the affected nodes. This is done by employing a circular curve fitting algorithm. Results indicate a high degree of accuracy in localizing a jammer has been achieved.

## Introduction

Wireless networks, in general, and wireless sensor networks (WSNs), in particular, are increasing in popularity due to the recent rapid decline of costs and the constant availability of new technology. Much research is also being done in terms of designing *green* protocols to make wireless networks energy efficient. As a result, wireless networks are now being extensively deployed from home automation systems to industrial communication systems. As these networks gain in scalability and size, the dependence on human manual intervention has to be minimized [1]. The networks have to be self-sufficient in all concerns especially in the aspects of self-configuration, self-organization, self-awareness, self-recovery and self-protection [2].

All wireless networks are vulnerable to physical security threats such as jamming and so is the case for WSNs as well [3]. Jamming is the deliberate use of radio signals in order to disrupt any sort of communication under particular radio frequencies [4]. Low power sensor nodes in WSNs can be significantly disrupted by the presence of a malicious external node transmitting in the same frequency channel. The interference of data packets and loss of communication heavily affects the availability security goal in a WSN [4]. Battery resources are wasted when the simple sensor nodes continue to function normally in terms of transmitting and sensing, without realizing the presence of a jammer. This waste, in turn, shortens their lifespan and the lifetime of the network. This waste occurs when nodes continue to be active in presence of a jammer without any benefit [5].

In addition to the interference, there will be an alarming number of collisions. Nodes with ability to detect collisions will try to re-transmit the failed packets continuously thereby spending further more energy in the process [6]. Thus, the impact of a jammer on WSNs may be considered more significant as compared to superior wireless networks because of the limited resources of energy, lower intelligence and deployment in remote locations. Furthermore, the wider the range of the jamming transmission, the more will be the number of jammed nodes. Therefore, a proper method to detect the presence of a jammer in the network is necessary. The estimate of an approximate location of the jammer needs to be determined so that further action can be taken to restore functional availability of the network. Furthermore, it will be highly beneficial if affected nodes could reduce their power consumption by stopping functionality temporary so that device energy can be preserved during the duration of the jamming attack.

The contribution of this paper is a partially autonomic scheme that detects and localizes a jammer using distributed sensor agents. It is an extension of an earlier work in [7] and employs the framework for self-configuration from Ashraf et al. [8]. Essentially, the goals of this paper are, 1) to detect the location of the jammer with good accuracy, 2) to develop an effective power saving algorithm for the affected nodes in the presence of a jammer, and 3) to implement the algorithm in a suitable network simulator.

## Related Work

### Jammer Localization Techniques

Jammers can severely disrupt the communications in wireless networks, and many schemes have been developed to localize and mitigate the effect of jammers. Jammer mitigation can be divided into hardware based and software based techniques. Hardware based solutions require specialized equipment which measure the transmission and reception properties of wireless signals. The basic principle is to generate a cancelling signal which mitigates the jamming threat. On the other hand, software based techniques can only be used to detect and localize the jammer, and cannot be properly used to cancel the effect of the jammer. Techniques such as frequency hopping and channel surfing constantly allow the system to switch frequency channels to avoid the jamming frequency. Centroid localization and weighted centroid localization techniques are also used for localization. Centroid localization works by collecting coordinates of the neighboring nodes, and calculating the average of the same. Centroid method is heavily dependent upon the network density. The weighted method enhances centroid based localization by introducing a metric to differentiate the nodes based on distance. Nevertheless, it is a problem in itself to accurately determine the distances between the nodes. However, accuracy can be improved by measuring Received Signal Strength (RSS) values can be utilized for acceptable levels of accuracy.

These schemes, however, do not consider reactive measures for the nodes under consideration. Reactive measures include changing the transmission behavior in the affected nodes or re-constructing routing tables. Reducing the duty cycle in the affected nodes is an interesting solution and it was found that none of the existing methods utilize the same. Our proposed scheme manipulates the duty cycles of the jammed nodes so that energy is not unnecessarily wasted for unsuccessful retransmission attempts. Authors of [9] focus on the detection and localization of a reactive jammer in an enterprise Wi-Fi network. The assumption is that the interference range of a target device is extended in the presence of a jammer. A centroid based algorithm is used to localize the jammer, and thus, the results of the algorithm depended on the number of all access points. The performance measurements included time needed to detect the jammer within certain accuracy. However no reactive measures are proposed by the scheme to counter the effect of the jamming, and no reduction in service levels was made.

Localization of a jammer is also discussed by [10]. They analytically prove that for multi-hop setups, the reception range of a node may shrink and its neighbor list may change when a jammer becomes active. This information is used to identify the nodes under attack and localize the jammer. The performance metrics include the effect of node density, the range of the jammer and the effect on the jammer's position in the network. However, the major flaw in this approach is the assumption that the jammed nodes can still transmit the information of their reception range. This assumption ironically means that in spite of being jammed, the nodes can still transmit information to other nodes. In addition, no measure was taken to reduce the energy consumption in the affected nodes.

The same authors [11] use the measurement of the strength of jamming signals to approximate the location of a jammer. They consider jammer localization as a non-linear optimization problem. The performance is studied by varying node density, jamming power, and number of jammers in a 300 square meter simulation layout. However, the proposed scheme does not take any steps to reduce the energy consumption levels in the affected nodes. In terms of accuracy, the scheme has the ability to perform better by including other non-measured factors.

Liu et al. [12] proposed a Virtual Force Iterative Localization (VFIL) algorithm that utilizes from the prior knowledge of network topology as well. One advantage offered is that unlike centroid based approaches, this algorithm is less sensitive to node densities. However, the scheme assumes that, after deployment, the affected nodes are stationary. In comparison, our proposed scheme considers mobile nodes, as well as a mobile jammer. In retrospect, a common assumption in both the schemes is that individual location coordinates are known by each node. In addition, two models are further developed by the same authors in [13] which are based on region and signal-to-noise ratio.

Babar et al. [14] presented the requirements for efficient defense strategies against jamming attacks focusing on complex methods such as using game theory and cross layered approaches. They also present a jamming model to help understand the sequential flow of events in a jamming attack. Our proposed scheme, in addition to jammer localization, takes further measures to allow for energy conservation in the network.

In their paper [15], authors proposed an algorithm called double circle localization. To evaluate the accuracy, they define localization error as the Euclidean distance between the estimated and the actual location of the jammer. They also make use of experiment based verification on MICAz motes in a square field of 10m by 10m. The transmission range is set at 3m, and the nodes are deployed within 2m of each other. However, the research work is restricted because of its inability to localize multiple jammers, as well as directional jammers. Furthermore, no further steps were taken to ensure energy efficiency in the presence of a jammer.

The work by [16] proposed to locate the mobile nodes under jamming attack using RSS based information. Not only do they localize a jammer, they also propose to localize a mobile node based on the jamming information. However, this approach doesn’t take any remedial measures to reduce the power consumption of the affected nodes also. In [17], authors succeed in achieving good performance while determining the locations of multiple jammers. However, this work fails to localize a single jammer, or multiple jammer with non-overlapping jammed areas. The strength of the method employed relies on the bifurcation of the multiple jammed areas. They assume nodes to be randomly deployed and static in nature. Node are assumed to know their own locations as well. This is not a new assumption and many existing applications require the network components to be aware of their respective locations [10].

The work in [18], on the other hand, is based on gradient descent minimization algorithm, which utilizes PDR information instead of the commonly used RSS values. The algorithm is implemented in a distributed setting and doesn’t require specialized hardware. One drawback is that this method cannot differentiate between jamming interference and legitimate packet drops due to congestion of the network. Another drawback mentioned by the authors is that there is a high sensitivity to local minima, i.e. the choice of the initial point can heavily affect the end result.

Readers are directed to the dedicated survey on jamming attacks to understand the patterns in the various countermeasures [19]. This survey, however, does not address the newer localization techniques in great detail. However, it serves as an excellent starting point for those interested in an overview on the jamming attacks. Readers are also recommended to go through [4] to understand how autonomic mitigation methods can be applied in the case of a jammer attack. Furthermore, it is worthwhile to highlight that jamming attacks are not restricted to the wireless world, but also significantly affect wired communications such as in optical broadband communications [20].

### Autonomic Self-Configuration

In traditional computing paradigms, self-star (self-*) refers to the set of self-organization, self-awareness, self-adaptation, self-design, self-building, self-repair and similar paradigms. The philosophy of self-* seeks to describe essential qualities that should constitute the behavior of an autonomic system. An autonomic system is one which is able to sustain itself completely and automatically. The automation does not lie in individual devices but is manifest as a property of the system itself [21].

**Fig 1:**
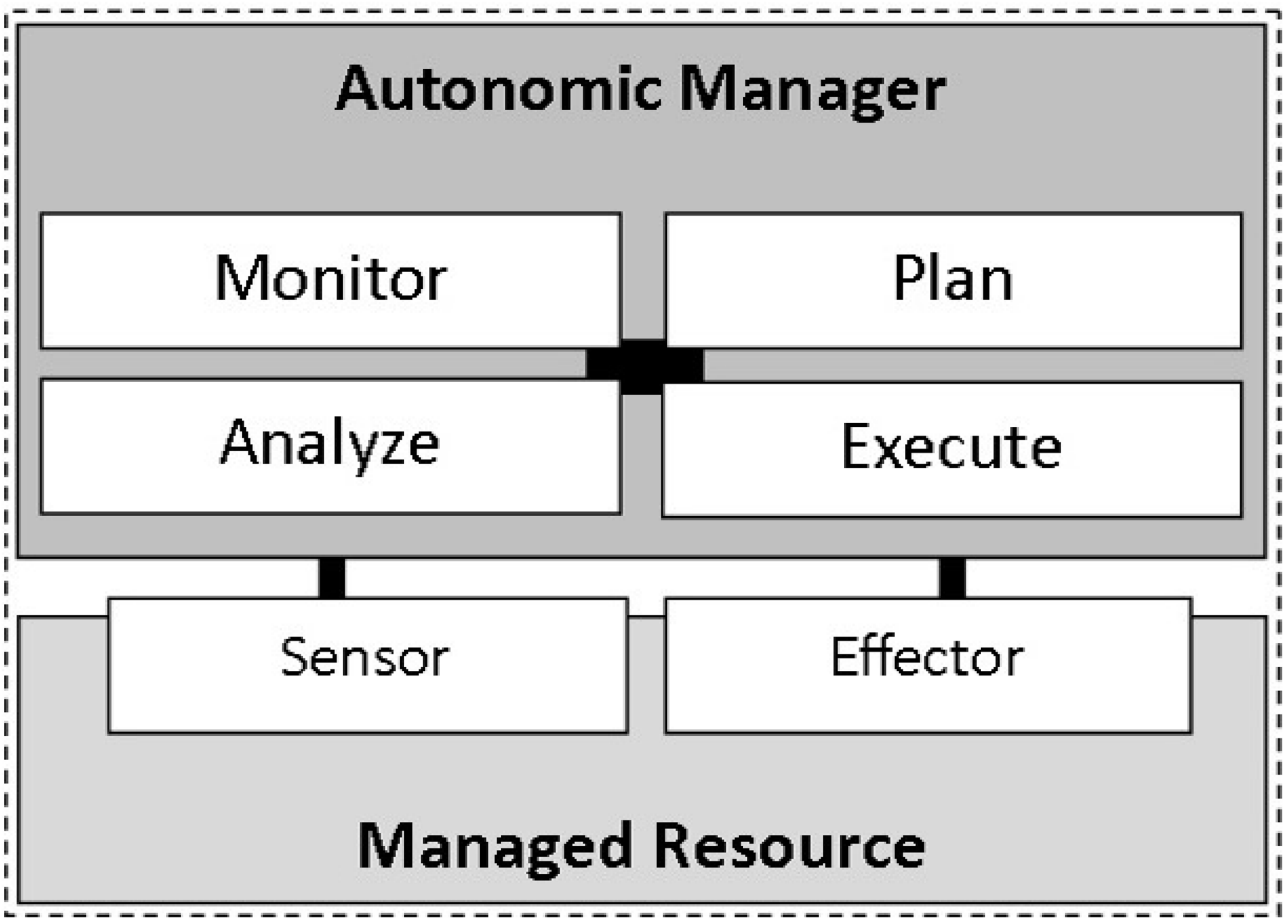
The autonomic control loop divided between an autonomic manager and a managed resource.

The MAPE control loop provides the foundation of autonomic computing [22]. It provides the definition of the essential functions that are needed in an autonomic system. Thus, adopting the theory of autonomic computing in the jammer localization algorithm means to follow the architectural elements suggested by the autonomic control loop: the elements to monitor, analyze, plan and execute. The MAPE reference model is presented in Fig 1. These four components have been discussed next on the basis of their functionality in the context of the jammer localization algorithm:

#### The Monitor Module

As the name suggests, this module is responsible for generating the input to any autonomic system, and essentially observes parameters of the environment. This allows the autonomic system to be aware about its state, and to observe any possible changes. Jamming detection can benefit from a wealth of parameters that can be monitored in terms of device states, environment states, and sensor as well as control data. Constant monitoring allows the nodes and the jammer to be mobile as well.

#### The Analyze Module

After the monitoring phase, the system is in possession of control and sensor data. Next, this data is analyzed in order to derive benefit for the autonomic system. The analysis stage is also adopted in the jammer localization algorithm. The main responsibility is to provide a mechanism that can analyze the different status of nodes in the network and classify them in two categories; one that has been affected by the jammer, and another, that hasn’t. A proper analysis thereby offers an advantage to the central node to understand the presence of a jammer.

#### The Plan Module

The plan module provides techniques to achieve a specific set of outcome, e.g. in our case to localize a jammer. The plan module works with higher level, user defined policies, rules and regulations which are basically system level constraints. To actuate any part of the system, planning is important as it considers the functionality of the system components.

#### The Execute Module

The execute module controls the execution of the formulated plan, and also offers feedback to the monitor module. It is responsible for handling the signaling to the actuators and sensors in an autonomic system. In our scenario, execution of the plan takes effect in the form of self-configuration.

## Autonomic Jammer Localization

### Network Model and Assumptions

Principally, the communication method assumed in this scheme is restricted to the media access (MAC) layer, for multi-hop and single-hop supporting protocols. In our simulation test, we implemented the network on ContikiMAC protocol with default contention parameters. The intrinsic performance of the MAC protocol does play an important part in obtaining proper results, especially when it comes to timing and collision based parameters. However, in this study, the effect of the MAC protocol on timing and collision is not being studied, as we primarily focus on the accuracy of detection of the jammer. Thus, we are more concerned about the messages reaching successfully to the recipient instead of how fast or how efficiently the message transfer takes place. Furthermore, the proposed localization solution is valid for the following category of networks and assumptions:

#### Multi-hop and single-hop

The algorithm works on either category of wireless networks. We assume the presence of a single central node, such as a gateway or a data-sink that can communicate with all the nodes in the network.

#### Location Aware

Each node in the network is aware of its location coordinates. This is not a new assumption and many existing applications require the network components to be aware of their respective locations [10].

#### Mobile and Stationary

Upon deployment, the location of nodes can change. However, we assume that each node is aware of its location, and can inform the central node upon change and update its new location coordinates.

#### Mobile Jammer Model

We assume a jammer that appears in the network to disrupt communication. Our scheme will also work with a mobile constant jammer which is continually emitting a radio signal, and changing locations in the process of doing so.

Omnidirectional

We assume a single jammer is equipped with a uniform omnidirectional antenna. It may or may not have the same range of transmission as the other nodes in the network. Either way, the localization algorithm is capable of calculating the jammer range.

#### Mobile Node

Nodes are capable of configuring their transmission parameters and of providing their location parameters to the central node.

#### Active Node

A node is active if it is unaffected by the jammer, and can transmit and receive packets from other neighboring active nodes.

#### Jammed Node

**Fig 2:**
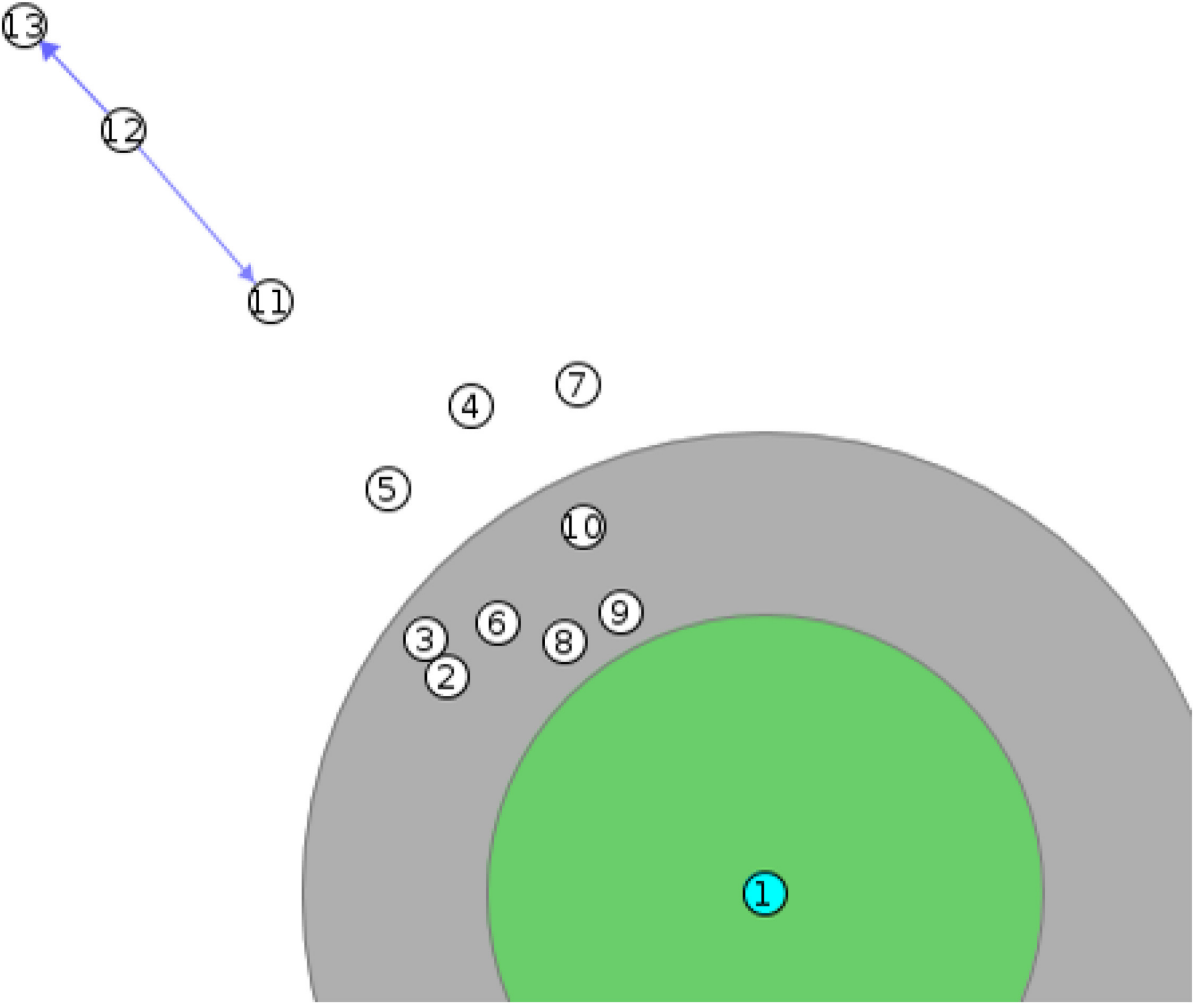
Network layout showing interference range of a jammer and the affected jammed nodes. Node 1 is the jammer. The internal circle signifies the transmission radius of the jammer, whereas the outer circle signifies the total interference range of the jammer. Nodes 11–13, 4–5, and 7 are not affected and can communicate between each other.

A node is considered to be jammed if it lies within the transmission radius of a jammer. Functionally, a node is jammed if it cannot receive or transmit any packets. A network layout showing an example of multiple jammed nodes is presented in Fig 2.

### Algorithm Design Challenges

To localize a jammer, two major questions need to be answered: 1) How to estimate the position of the jammer, and; 2) how to estimate the transmission range of the jammer? In addition, another question that needs to be answered is which nodes should participate in the energy conservation process?

The main idea of our localization method to estimate the position of a jammer lies in the fact that a jammer location can be determined by checking the location coordinates of the affected nodes. Using that information, we can determine, with a high probability, an approximate affected circular area of a jammer.

All entities with wireless transmission capability have a limited range of spherical communication. In our case, for objects on planar surfaces, it is safe to assume two-dimensional circular transmission with the node at the focus. We are assuming a large scale circular WSN whose total range exceeds the range of the jammer by a significant amount. To estimate the transmission range of the jammer, we make use of the fact that all jammed nodes will lie within a circular area of a given radius. Nodes which are unaffected will lie outside this circle. Essentially, this becomes a geometric problem to find a best fitting circle for a set of points on a planar surface.

Nodes that participate in the energy conservation process have to fulfill the following criteria. 1) They have not received any packet from a neighboring node for a given time interval; 2) they have not received a periodic message from the central nodes in the said time interval; and 3) they have not received any acknowledgement packet for their transmitted packets to other nodes.

### Overview of the Proposed Algorithm

**Fig 3:**
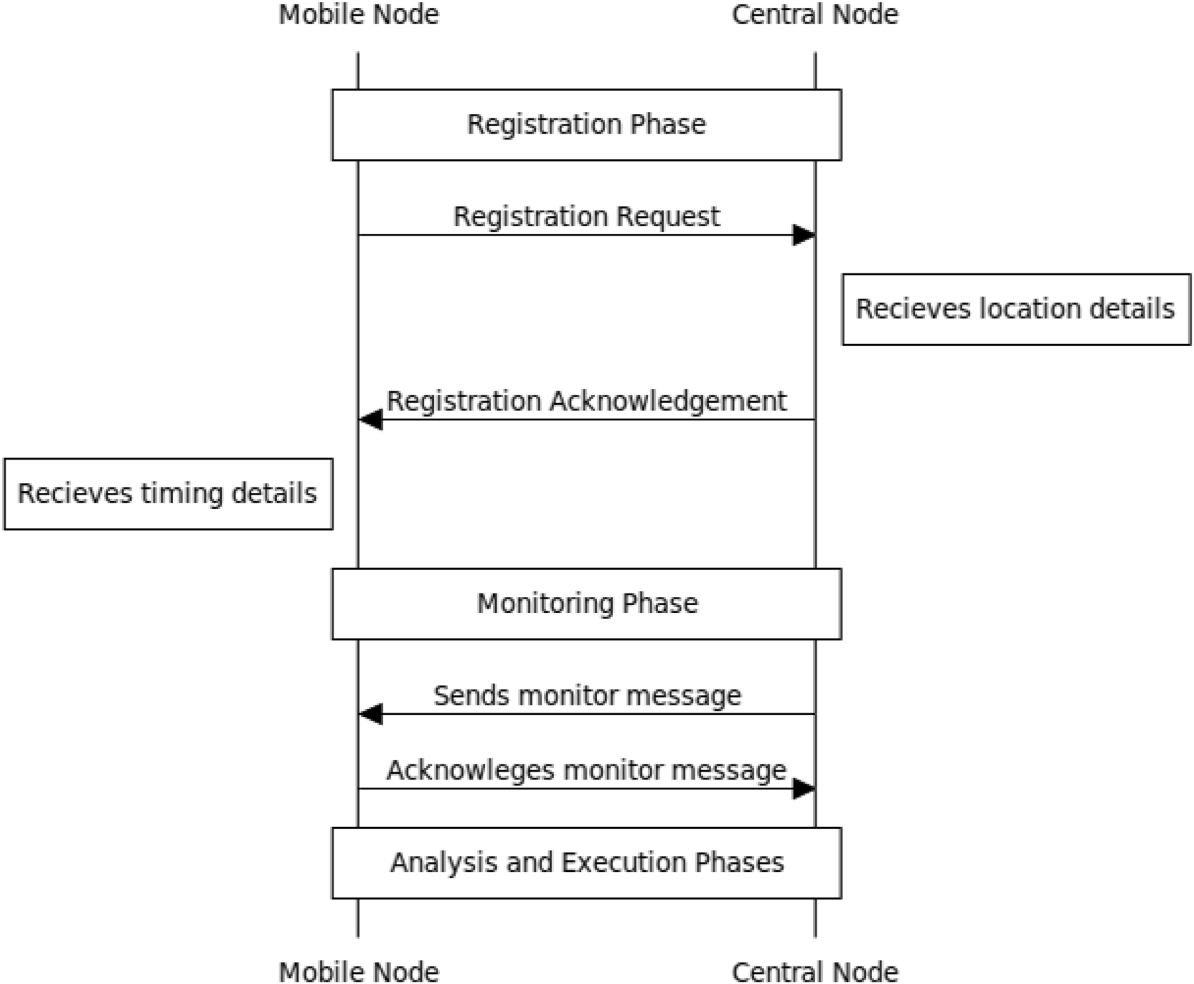
Message sequence flow between a mobile node and a central node.

The central node is able to detect the presence and approximate the location of the jammer with the help of the other nodes in the network. In the mechanism, the central node transmits signals to each of the mobile nodes at predetermined intervals and receives a return acknowledgement from each of the mobile nodes. Any absence of acknowledgement from any of the mobile nodes corresponds to a detected jammer. Obviously, the whole algorithm builds on the assumption that the mobile node is unable to return the acknowledgement because of its close proximity to the jammer. The general flow of this operation for the method is as follows, and has been illustrated in Fig 3:

Step 1: When a mobile node first enters a network, it will be registered and configuration of control values will take place. As an example, a broadcast based registration technique could be employed [23]. This also represents the plan stage of the autonomic control loop. In this stage, the mobile node also sends its location information.
Step 2: The central node will routinely send a message to the nodes at a certain time and the nodes will acknowledge the message. This represents the monitor stage of the autonomic control loop.
Step 3: If the acknowledgement message is not received back at the central node, then the central node will establish a list to keep track of the affected nodes and then approximate the location of a possible jammer. This represents the analysis stage of the autonomic control loop.
Step 4: If the mobile node doesn’t receive any of the expected routine messages from the central node, then it will assume presence of a jammer within its proximity and will self-configure its energy conservation method for a specific amount of time. This represents the execute phase of the autonomic control loop, which essentially is the property of self-configuration.

When a mobile node first enters a network, it requests to join the network. The central node responsible for the management of the network replies by acknowledging and sending an available identification number for the mobile node that will be used in subsequent communications. Once the registration with the central node is done, mobile nodes send their current location coordinates to the central node. The location coordinates may be exact GPS coordinates or approximate coordinates derived from various localization techniques such as range-based or range-free triangulation method. The accuracy of this scheme in part depends on the accuracy of the location information of the mobile nodes. The details of registration request, transfer of control values and location parameters is out of scope of this paper, and the self-configuration framework and protocol in [8] is assumed to handle the communication.

Once the central node receives the location information during the registration stage, it sends values for three timing parameters: T_wait_, T_ping_ and T_sleep_ to the nodes and also updates the network node list and node count. The three timing parameters have been defined below:

- From a node’s perspective, T_wait_ refers to value of time when the next monitoring phase is scheduled to take place. This is necessary because a node may join in anytime and it is required to know the time of the start of the next monitoring phase when it has to constantly listen for incoming ping packets. This value sent for this variable depends on the time of registration of the new node. Its value is always less or equal than T_sleep_. Since nodes are assumed to be unaware of a global time, this helps to synchronize the nodes.
- T_ping_ refers to the total duration of time a node has to listen for messages at the start of the monitoring phase. For simplicity, this value is constant for a network at all times. As a result, all nodes in the network will listen simultaneously to incoming messages. If a node does not receive a message during this time, it will sleep after the end of this period.
- T_sleep_ is the time left till the next monitoring phase after duration T_ping_ has ended. It signifies the interval at which the monitoring phase is repeated. Consequently, a node which hasn’t received a ping in the preceding monitoring phase will sleep for this amount of time.

For simplicity, T_ping_ and T_sleep_ are assumed to be constant for a network. But if central control of these parameters is required then the central node can update all nodes with any new values, with any suitable technique of reconfiguration.

### Locating the Jammer: Monitoring and Analysis

The algorithm for localizing the jammer can be summarized as follows:

Step 1: The locations of the unreachable nodes can be represented as a set of <x, y> coordinates on a plane. The central node will be needed to compute a best fit circle through these set of points. Many methods exist in geometry which allow for fitting circles to a given data. Five of them are discussed by Umbach and Jones in [24]. For our purpose, we utilized the method and algorithm provided at [25].
Step 2: After obtaining focus point (x_f_, y_f_) and the radius length (l_r_) of the best fit circle, the central node will next calculate the distance between all the affected nodes and the newly calculated focus point.
Step 3: The central node will obtain the average of these distances which we term as D_av(x, y)_.
Step 4: Subsequently, the central node will remove any outliers by comparing the D_av(x, y)_ with the actual individual distances from the focus point to all the affected nodes. This is due to the fact that distance from any affected node to the calculated center must be less than or comparable to the average. Any large value would mean a far-away location of the node, and would thus signify the presence of an outlier. An outlier can heavily affect the accuracy of localization, and removing its influence can greatly enhance the accuracy. Such an outlier will be removed by comparing its distance to the center and the average distribution of distance. Any extreme value that is more than double the D_av(x, y)_ infers either a wrong reading or a malfunctioned node with no presence of a jammer.
Step 5: With the new set of points without the influence of an outlier, the central node will re-calculate the center and the radius of the best fitting circle.

These four steps have been presented in Table 1. Few cases arise out of this. In the first case, the affected nodes are located along the border of the wireless network, and therefore it can be safely assumed that the jammer is also located near the edge of the network. In the second case, the group of affected nodes lies inside the network with perfectly working nodes scaling the border of the network. In the first case, the jammer may be either located between the affected nodes or outside the network area with some of its jamming range interfering in the network area. In the former case, all of the transmission of the jammer will be responsible for the interference, whereas in the latter case, the interference will be caused by a part of the jammer transmission. In the former case, the jammer will have a high probability of being around the new calculated center as the interference is assumed to be inside the circular jammer radius of transmission. Another case that can crop up is if only one or few nodes are found to be unreachable and all other nodes reply to the central node’s messages perfectly. In this context, either the individual nodes are down or the jammer doesn’t have much range. There is a high probability that nodes have malfunctioned or ran out of energy.

**Table 1:** Algorithm for locating the jammer using *n* unreachable nodes.

**if** *monitoringPhase* = = 1 **then**
array[*x*_*n*_, *y*_*n*_] ← <*unreachable nodes*>
[*x*_*f*_, *y*_*f*_, *l*_*r*_] ← computeBestFit(array[*x*_*n*_,*y*_*n*_])
array1[*d*_*n*_] ← compDistance(array[*x*_*n*_,*y*_*n*_], *x*_*f*_, *y*_*f*_)
D_ay(*x*,*y*)_ ← summation(array2[*d*_*n*_]/ *n*)
**if** array1[*d*_*n*_]> = *2*D_ay(*x*,*y*)_ **then**
array2[*x*_*n*_, *y*_*n*_] ← remOutlier(array[*x*_*n*_, *y*_*n*_], *n*)
**end if**
[*x*_*f*_, *y*_*f*_, *l*_*r*_] ← computeBestFit(array2[*x*_*n*_,*y*_*n*_])
**end if**

### Self-Configuration: Planning and Execution

During the monitoring phase, nodes will generally stop sensing and transmitting and only go into listening/receiving mode. They will only switch on transmission to send the acknowledgement message after they receive the monitoring message from the central node.

In the event of not receiving a monitoring message, a node will go into a sleep mode for a duration specified by T_sleep_ parameter thereby saving energy. If these nodes had continued to function during this time in the presence of a jammer and attempted to transmit and re-transmit all failed messages, then a lot of energy would have been unnecessarily consumed.

In addition to the localization function, the proposed scheme also allows the central node to constantly monitor the status of the network by maintaining a list of registered nodes as well as store the information about their location coordinates. Furthermore, each time the node wishes to leave the network, it has to appropriately inform the central node and the latter has to acknowledge the request. The central node then updates the list of currently registered nodes. This ensures that the scheme doesn’t confuse non-reachable nodes with nodes that have left the network.

## Performance Evaluation

In this section, we highlight our evaluation results on the performance of the proposed localization algorithm. The scheme was simulated on COOJA simulator in Contiki OS using Zolertia Z1 nodes. In our simulation test, the communication between the Zolertia Z1 nodes was executed on ContikiMAC protocol with default contention parameters that are provided in the simulation environment. The choice of the underlying MAC protocol and the associated performance plays an important role in reducing the number of collisions as well as inefficient message delivery. However, studying the effect of the underlying MAC protocol on timing and collision is out of scope for this research, as the results primarily focus on the accuracy of detection of the jammer using the proposed scheme. The Contiki OS uses ContikiMAC protocol as the default mechanism for effective power-saving (used for execution in self-configuration) in which the sensor nodes can make their radio listening ability to be turned off when not required. Although the ContikiMAC is a power-efficient protocol, it still requires accurate timing and contention between successive transmissions. The ContikiMAC protocol makes use of Clear Channel Assessment (CCA) which measures the Received Signal Strength Indicator (RSSI) of the radio transceiver to alert the availability of the radio channel. ContikiMAC allows decreasing the power consumption of the sensor nodes by switching off the radio when not necessary. A proper duty cycling algorithm becomes the key for power-efficiency, the functionality of which we exploit in the self-configuration part in the proposed scheme.

The simulation parameters used are listed in Table 2. The simulation parameters that are described for the simulation environment include area size, default transmission distance of the nodes and the jammer, default distance between jammer and central node, default density of nodes, as well as the values employed for t_ping_ and t_sleep_. The choice of values for t_ping_ and t_sleep_ don’t affect the localization algorithm, but contribute to the overall detection time.

**Table 2:** Simulation Parameters.

Components	Description
Model	Zolertia Z1 WSN mote
MCU	MSP430F2617 16-bit RISC CPU@16MHz
RF transceiver	CC2420 2.4 GHz @250kbps Spreading gain: 9dB 128(RX) + 128(TX) byte buffering
Identification	Contiki RIME Address
Embedded OS	Contiki
Simulation Area	200m x 200m
T_ping_	5s
T_sleep_	15s
Simulation Runs	20
Default distance between jammer and central node	60m
Default transmission range of nodes and jammer	50 m
Default density of nodes	20 nodes (Circular)

We can compare accuracy of detection for few criteria with the localization results of “*range-free and energy efficient localization approach using few mobile anchor nodes*” (RELMA) obtained recently in [26]. The intrinsic difference between the two schemes disallows for an apple to apple comparison. Their research work presents results related to localization energy consumption in RELMA, and average localization error in the scenario of varying number of devices, sensing range as well as the velocity of the mobile nodes. Localization accuracy is defined as the difference between the actual location and the calculated location. With a transmission range of 50 m setting other parameters at default (see Table 2), our scheme results in 89.03% accuracy in localization i.e. with an error of 5.48 meters. Results for RELMA [26] suggest accuracy ranging between 7–20 meters for a sensor layout of 100 m with range 50 meters. For a sensing range of 18 m, the average localization error in [26] stands at around 10 meters. Furthermore, the ratio of sensing range to interference range is kept constant at 2 for our scheme, whereas [26] vary the ratio from 1.5 to 4.5. However, we do fluctuate the jammer’s transmission radius which essentially increases the total range of interference, and thereby effects more nodes. Also, our proposed scheme exhibits better performance than sink at the origin approach (SOL) [27] where the measurement accuracy goes up to 80 meters for a layout of 100m x 100m. That illustrates an accuracy of around 20% using [27] for a comparable sensing range (50 meters).

**Fig 4:**
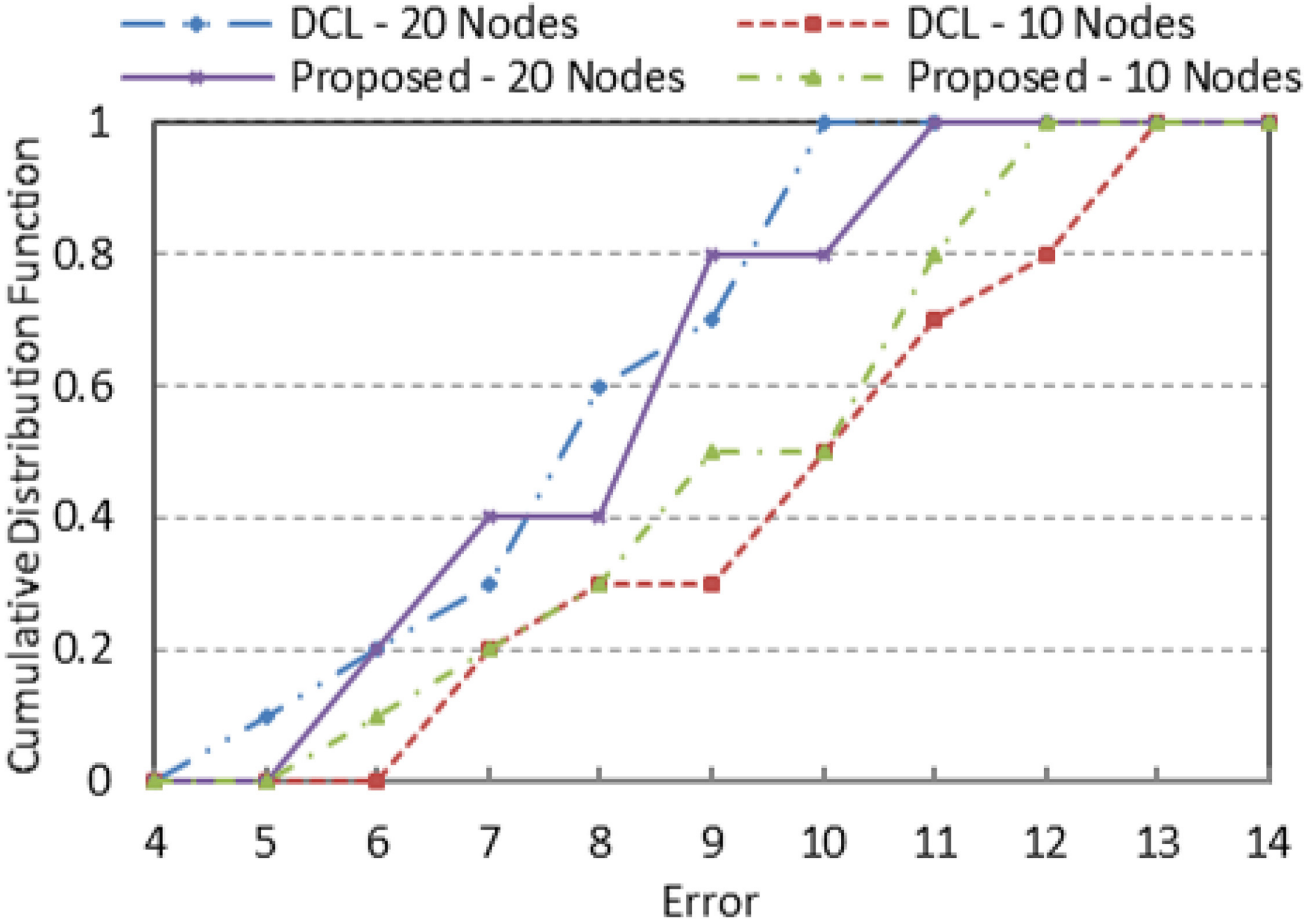
The impact of density of participating nodes on accuracy of jammer localization.

We also compare part of the accuracy performance of our proposed scheme with double circle localization algorithm (DCL) proposed in [15]. The results for cumulative distribution function for localization error were adapted from [28]. The comparison between the proposed scheme and DCL is illustrated in Fig 4 for N = 10 and 20 for a transmission radius of 50m. The average localization error is about 8 m, and it decreases as the number of nodes increases to N = 20. Trend wise, the cumulative distribution function is very similar between the two methods.

### Impact of Jammer’s Position

**Fig 5:**
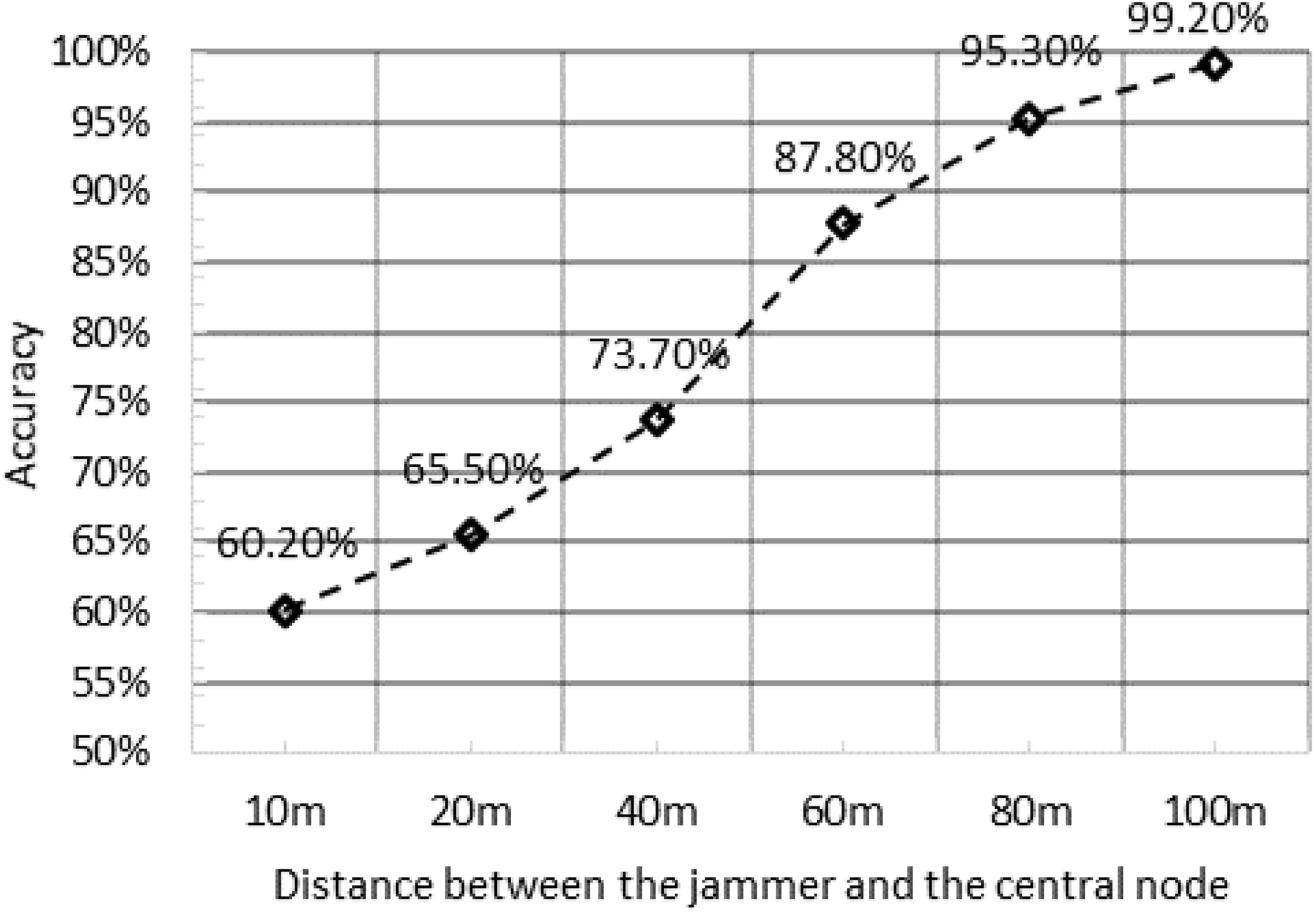
The impact of jammer’s position in the network on accuracy of localization.

We placed the jammer at varying distances from the central node, and plotted the accuracy of the algorithm when setting the jammer range to 50 m and number of nodes to 20. We observed that when the jammer is located far away from the central node, the accuracy of localization is very high at around 98%. However, when the jammer is brought closer to the central node, then the errors in localization start to increase. The accuracy reduces significantly when the jammer is brought within 10m of the central node. This increase in estimation errors is due to the presence of the jammer that affects the wireless transmission in the central node itself. This eventually causes the central node to assume all other nodes are affected by the jammer as it can’t communicate with any node in the range at all. Thus, in effect, assuming a circular distribution of nodes, the central node localizes the jammer location to be near to itself and the accuracy would have been quite high. However, an even circular distribution wasn’t considered in the simulation and nodes were randomly scattered leading to such a low accuracy. The results are plotted in Fig 5.

### Impact of Jammer’s Range

**Fig 6:**
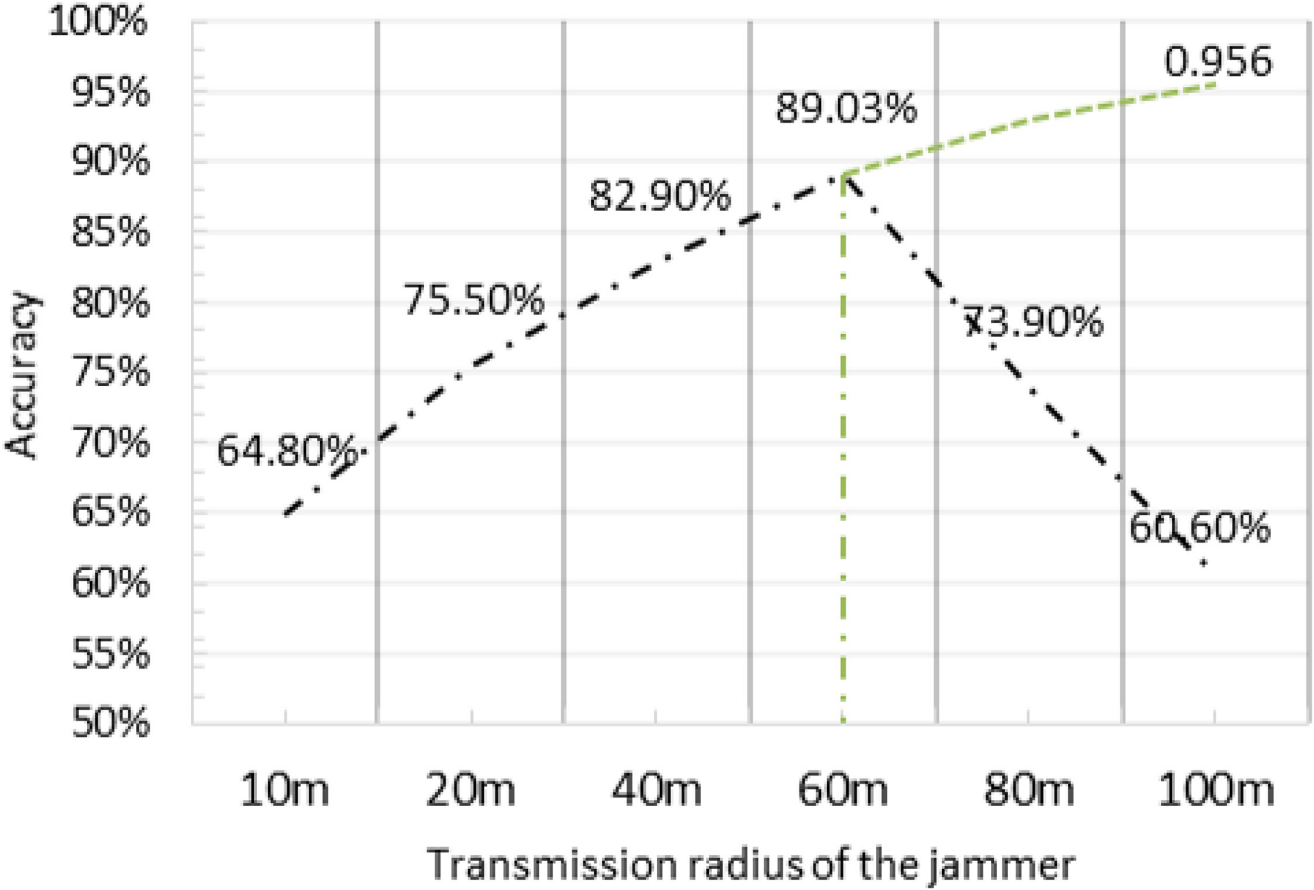
The impact of jammer’s transmission range in the network on accuracy of localization.

We measured the accuracy of a 20 node network when changing the jammer’s radius from 10m to 100m, and plotted the results in Fig 6. The jammer was kept 60m away from the central node. As the jammer’s transmission range increases, the accuracy of localization improves at first but then decreases significantly. The accuracy gets better when the transmission range of the jammer is increased from 10m to 60m. However, any additional increase steeply affects the accuracy, as can be seen in Fig 6. The initial increase is due to the fact that the jammer will be affecting more and more number of nodes with increasing jamming range. This provides additional data points of affected nodes to the localization algorithm, which intrinsically produces better results with more data points. The decrease at higher transmission range is attributed to the fact that the jammer will be influencing the central node itself due to such a high range of transmission, hence, reducing the central node’s signal to noise ratio.

### Impact of Node Density

**Fig 7:**
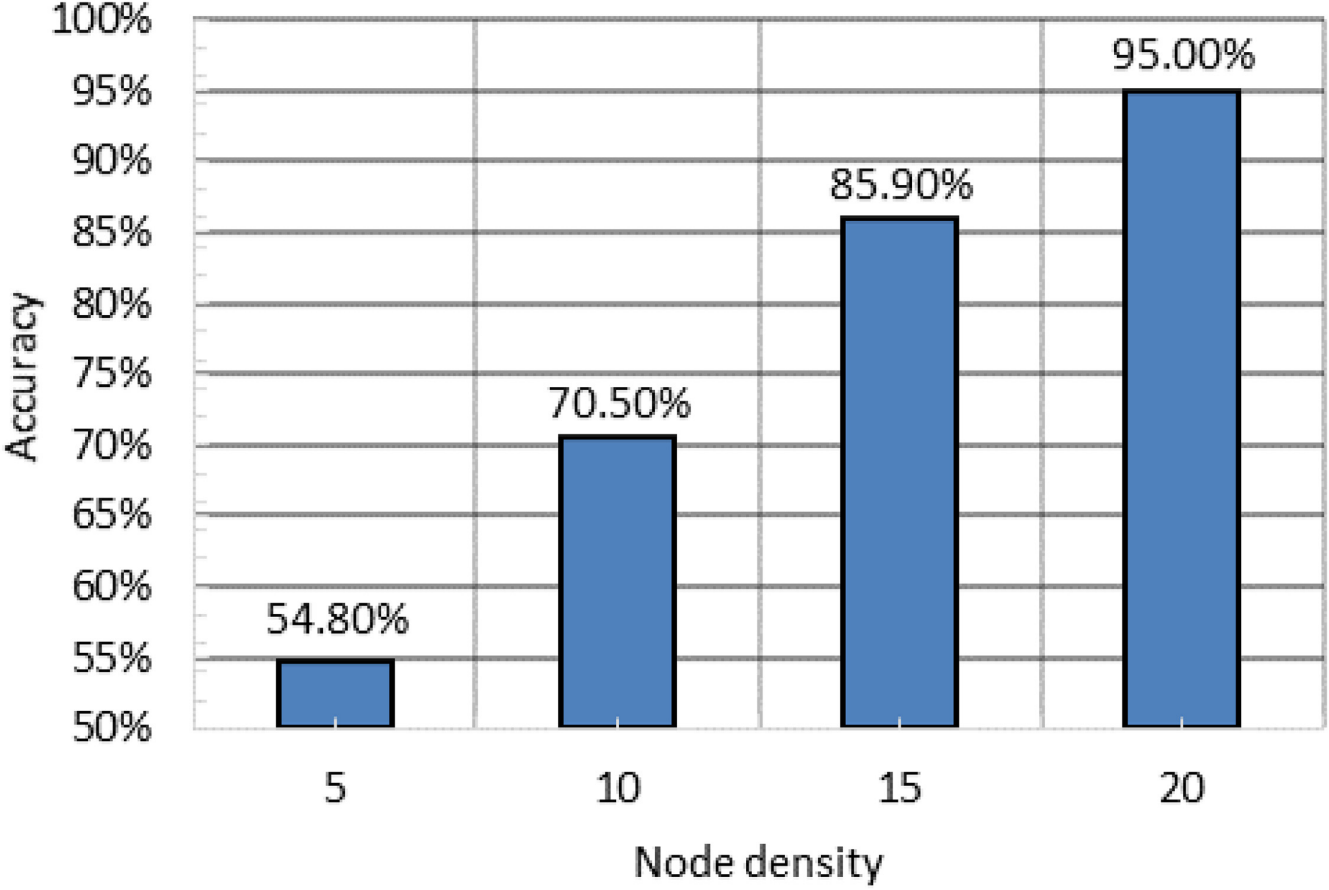
The impact of density of participating nodes on accuracy of jammer localization.

We investigated the impact of the node density on the localization algorithm by varying the number of nodes from 5–20, while having fixed the jammer range to 50m at a distance of 60m away from the central node. This is done to isolate the effect of jammer on central node from the effect of jammer on participating active nodes. We plotted the accuracy of localization vs. the density of the participating nodes in Fig 7. It can be seen from the figure that as the number of nodes increase, the performance of the localization algorithm improves. A higher nodal density provides additional data points of affected nodes to the localization algorithm, which intrinsically produces better results with more data points.

### Power Consumption

Power consumption is one of the major concerns in WSN devices. A majority of the sensor nodes are battery powered and efficient energy management allows to lengthen the timespan of the network as a whole. Lately, research is more focused on power efficient communication as device to device communication consumes more power than computational processes in the device itself. Other ways of efficient power management include effective topology control, efficient routing protocols and resourceful radio medium access methods.

For the accurate evaluation of the self-configuring power-saving mechanism, we also studied the effect of duty cycle on the power consumption. The power-saving via self-configuration works by reducing the idle listening, and computational time on the effected nodes, after the presence of a jammer has been confirmed. This indirectly leads to reduce collision events as well as overhearing. The tradeoff is a slight increase in the messaging overhead and the complex mechanism that arises in synchronizing the timing schedules for the nodes. The power profiler feature in Contiki enabled the measurement of power consumption in the end nodes. The measurement method in Contiki multiplies the active time of each sensor node with its current consumption according to datasheet of each hardware. The following metrics are used for calculation:

The following metrics are presented for energy consumption:

1. Low Power Mode (LPM): LPM refers to overall power consumption in the low power mode, also known as the sleep mode where not much activity takes place inside the sensor node. Radio activity is completely cut off.
2. Central Processing Unit (CPU): CPU section of the graph shows consumption of energy by CPU based activities.
3. Radio Transmit: It is the amount of energy spent overall for total radio transmission.
4. Radio Listen: It is the amount of energy spent in the radio listening mode.

**Fig 8:**
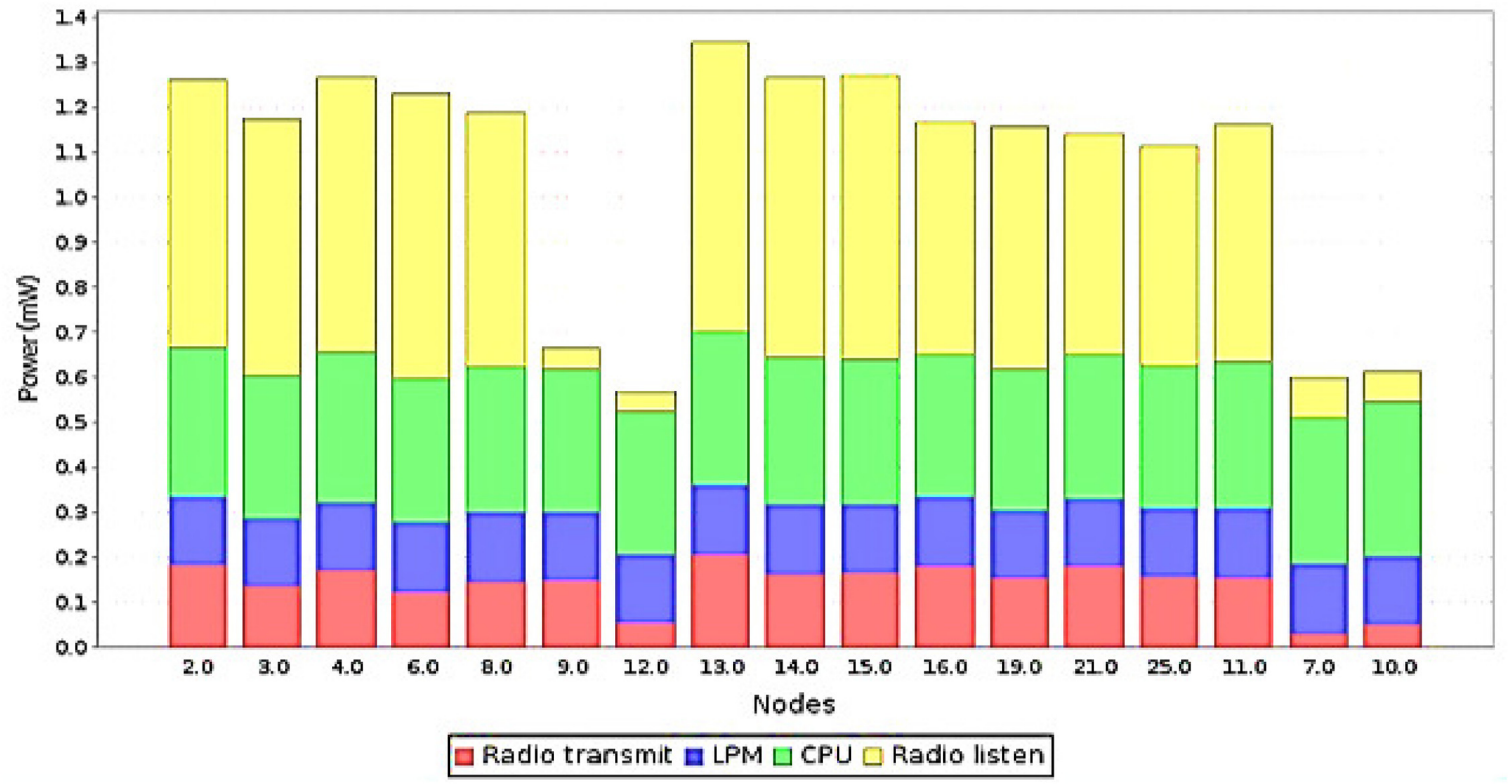
Energy measurements measured in mW for 25 simulated nodes.

Fig 8 highlights the difference of energy consumption in the execution phase of the self-configuration mitigation. A breakdown of the energy consumption measurements reveals the biggest factor in energy consumption. Affected nodes 9, 12, 7 and 10 have self-configured their duty cycle and stopped all communication, whereas other nodes continue to function as normal. The energy saving can be further be understood as a qualitative function of reduced duty cycles. Thus, a 50% duty cycle will result in 50% energy saving. Since the energy saving is not being claimed in the processing part of the jammer localization, therefore it is very hard to compare the same with other methods. In fact, what is claimed is the mitigation method, the steps that the network can take in the presence of a jammer to try to optimize the working. Clearly, the presence of a jammer will affect availability, therefore the best measure is to stop communication and save precious energy resources.

## Discussion

**Fig 9:**
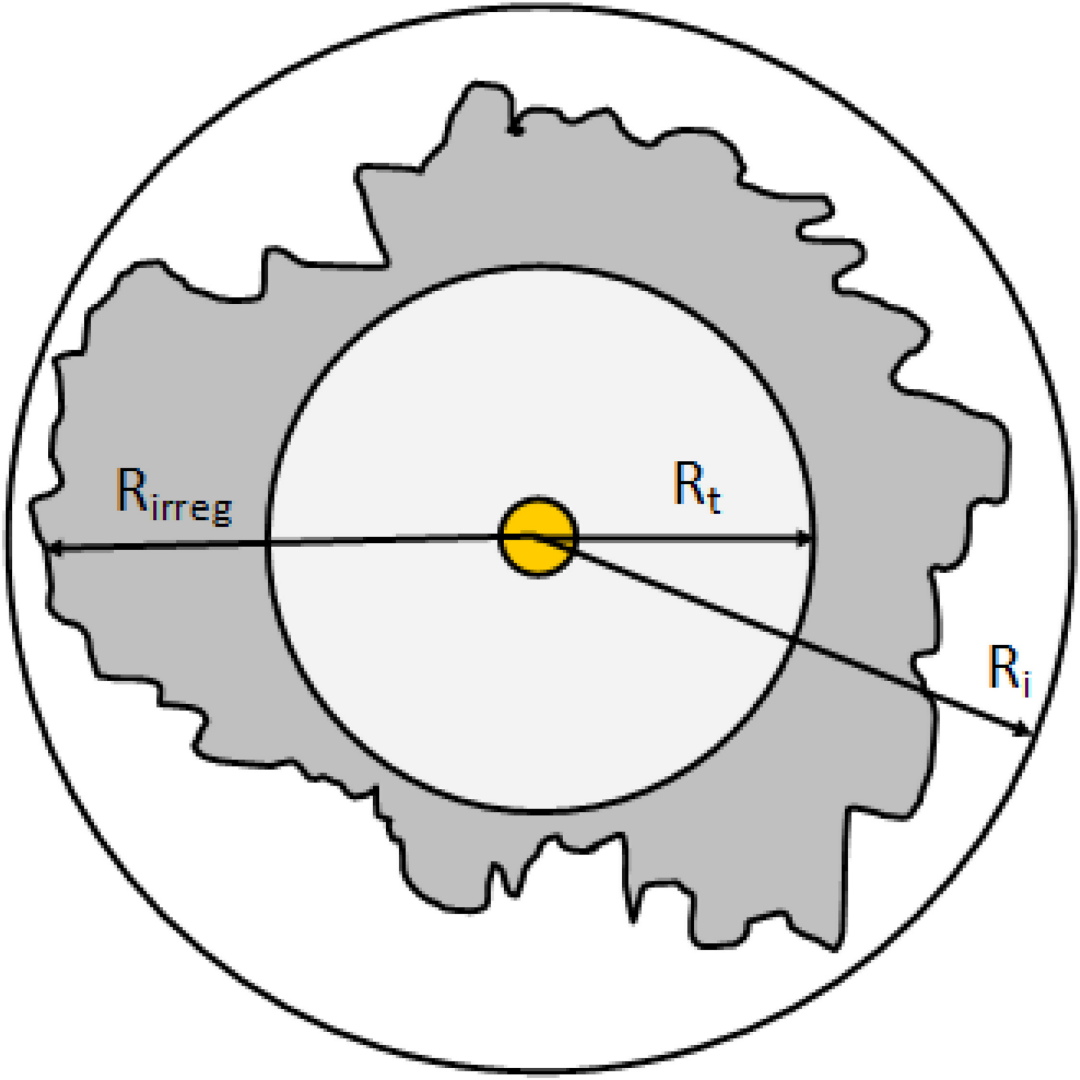
Irregular radio pattern.

Assuming a simple circular areas for wireless coverage is an acceptable starting point, however, actual wireless communications never match such ideal cases due to many factors such as antennae and environmental factors. The sensing and transmission range, (R_t_) of a node is much shorter than the communication and interference range (R_i_). Since the localization scheme makes use of R_t_, therefore, it can be argued that the scheme is also expected to provide an accurate localization for an irregular radio pattern such as illustrated in Fig 9. The ideal radio pattern for a jammer was presented in Fig 2. In our simulation study using the Contiki OS, we selected ‘Unit Disk Graph Medium (UDGM): Distance Loss’ model as the radio model. In this model, only the transmission distance of the nodes as well as the transmission success ratio can be configured.

The proposed jammer localization can be used in many applications including but not limited to health monitoring, home security, battlefield surveillance, and vehicular ad-hoc networks. Sensors are increasingly being used in a wide variety of applications, as they can be deployed randomly, accessed remotely and configured automatically. The proposed scheme will exist as one of the functions in such sensor devices and offer the ability to locate the presence of a jammer.

The energy saving via self-configuration can be qualitatively understood because of execution and implementation of reduced duty cycles. A 50% duty cycle decrease will result in more energy saving than a 20% decrease in duty cycle. The energy saving is not being claimed in the processing of the jammer algorithm such done in [26]. Instead, the mitigation method of self-configuring the duty cycle is being claimed to make the network more energy efficient, in the presence of a jammer. This is done in order to optimize the working of the network. Clearly, the presence of a jammer will affect the availability and communication, therefore the best measure in such a scenario is to stop communication in the affected nodes, and save precious energy resources. Similar to the observations by [18], areas around the jammers exhibit low PDR which results in the device to continuously re-transmit the message until an acknowledgement is received.

Previously, centroid localization techniques, and techniques employing the information from measured RSS values are used for localization. Centroid localization works by collecting coordinates of the neighboring nodes, and calculating the average of the same whereas RSS based techniques rely on the fact that RSS values change significantly with the distance from the jammer. RSS techniques are more popular because of their acceptable levels of accuracy. All localization techniques, however, do not take any further action after the localization is complete. In our method, we follow up the localization process with a self-configuration phase to reducing the duty cycle in the affected nodes. Thus, the self-configuration phase manipulates the duty cycles of the jammed nodes so that energy is not unnecessarily wasted for unsuccessful retransmission attempts. The proposed method was designed by including features from the autonomic computing control loop. In the past, some other techniques have utilized similar methodologies. For instance, wireless sensor continuously collect details about RSS values in [16] which can be considered to be a part of the “Monitor” phase of the MAPE control loop. These details are compared with known patterns and extreme values for specific parameters, such as an abnormally high RSS value in a specific frequency of the spectrum. In the technique presented in [3], the monitoring phase utilized the hearing range of the wireless nodes instead of using RSS values. In our case, we constantly monitored the devices by the routine messaging and feedback analysis. For “Analysis” phase, previous works have used game theory approach [14], geometric approach [15], knowledge of network topology [12]-[13], and optimization approach [11]. We employed a geometric approach for computing the best-fit circle for a set of points. A previous work [17] also employed geometric methods which employed the bifurcation areas of multiple jammers to localize the jammers. However, one major difference between our method and this approach is that our method can localize a single jammer but not multiple jammer, whereas the approach in [17] is able to localize multiple jammer but not a single jammer. With the assumption that the radio pattern of a jammer is perfectly circular, we managed to obtain satisfactory results. In addition, we followed up the localization technique with a self-configuration phase where the affected devices go to sleep and, therefore, conserve energy. Such a mitigation strategy is not included in many related works as their core focus is the technique for jammer localization such as in [9]. To complete the functionality of MAPE control loop, an execution phase in our work was necessary. On the other hand, some related works has attempted to mitigate the jammer threat by cancellation.

Localization accuracy is improved with increase in the jammer range similar to what has been found in [17]. Since a larger jammed area will result in more devices being jammer, therefore the number of data points inputted to the algorithm increase. Thus, there is more information available about the jammer position which increases estimation accuracy. However, we also found that increasing the jammer range further deteriorated the accuracy significantly. The decrease at higher transmission range is attributed to the fact that the jammer will be influencing the central node itself due to such a high range of transmission, hence, leading the central node to assume that all devices in the network are jammed. In such a situation, the jammer location will be estimated to be near that of the central node.

Work in [11] uses RSS values whereas we utilize the basic knowledge whether a device has been jammer or not. One advantage of our method over [12] is that our devices can be dynamic whereas their work assumes the devices to be static. A similar assumption exists in [17]. However, our method is highly sensitive to device densities when compared to the relatively less sensitive method in [12]. In summary, the proposed localization approach has the following characteristics.

1. Works well for localizing jammer in sensor area networks.
2. Energy efficient, and accurate method which uses network connectivity to determine presence of a jammer.
3. Also works in irregular radio patterns as in practical cases.
4. Trades-off among accuracy, complexity and energy-efficiency.

## Conclusion

This paper proposed a scheme to locate a jammer, and consequently to save energy of the affected nodes in the jammer affected areas. The jammer made use of the autonomic control loop, by monitoring and analyzing the locations of the affected nodes. The autonomic components of monitor, plan, analyze and execute were utilized for the design of the scheme. Constant monitoring of the device state through routine communication allows the nodes and the jammer to be mobile. Analysis of the communication pattern allows the central node to understand the presence of a jammer using a circular curve fitting geometric algorithm. We observed that 95% localization accuracy was achieved for a set of 20 nodes, when the transmission distance and actual distance of a jammer from the central node are similar. In the meantime, the affected nodes also monitor the area and execute an energy saving process upon failure of communication. Having planned the self-configuration scheme, the nodes can execute a lower duty cycle if the presence of a jammer has been established.

## Supporting Information

S1 File This is the data file containing three data sets. (XLSX)
